# GabiPD – The GABI Primary Database integrates plant proteomic data with gene-centric information

**DOI:** 10.3389/fpls.2012.00154

**Published:** 2012-07-09

**Authors:** Björn Usadel, Rainer Schwacke, Axel Nagel, Birgit Kersten

**Affiliations:** ^1^ Max Planck Institute of Molecular Plant Physiology,Potsdam, Germany; ^2^ Department of Botany, RWTH Aachen University,Aachen, Germany; ^3^ IBG-2: Plant Sciences, Institute of Bio- and Geosciences, Forschungszentrum Jülich,Jülich, Germany; ^4^ Department of Genome Research, Institute of Forest Genetics, Johann Heinrich von Thünen Institute,Großhansdorf, Germany

**Keywords:** plant, proteomics, phosphoproteomics, 2DE images, protein microarrays, gene-centric, data base, MapMan

## Abstract

GabiPD is an integrative plant “omics” database that has been established as part of the German initiative for Genome Analysis of the Plant Biological System (GABI). Data from different “omics” disciplines are integrated and interactively visualized. Proteomics is represented by data and tools aiding studies on the identification of post-translational modification and function of proteins. Annotated 2D electrophoresis-gel images are offered to inspect protein sets expressed in different tissues of *Arabidopsis thaliana* and *Brassica napus*. From a given protein spot, a link will direct the user to the related GreenCard Gene entry where detailed gene-centric information will support the functional annotation. Beside MapMan- and GO-classification, information on conserved protein domains and on orthologs is integrated in this GreenCard service. Moreover, all other GabiPD data related to the gene, including transcriptomic data, as well as gene-specific links to external resources are provided. Researches interested in plant protein phosphorylation will find information on potential MAP kinase substrates identified in different protein microarray studies integrated in GabiPD’s Phosphoproteomics page. These data can be easily compared to experimentally identified or predicted phosphorylation sites in PhosPhAt via the related Gene GreenCard. This will allow the selection of interesting candidates for further experimental validation of their phosphorylation.

## INTRODUCTION

Over the last few years we have witnessed the “coming of age” of many “omics” technologies in the plant field. This has led to valuable resources in the field of transcriptomics (Zimmermann et al., 2004), metabolomics (Tohge and Fernie, 2009), and last but not least proteomics (Schulze and Usadel, 2010). Moreover new “omics” disciplines spring to life, e.g., “fluxomics” (Schwender, 2011) or “enzymomics” (Gibon et al., 2004).

Transcriptomics data has been mined extensively using not only simple differential expression analysis but also correlation approaches which have led to a better understanding of many different processes such as starch metabolism or cell wall biosynthesis (Usadel et al., 2009a). However, it is significantly more difficult to integrate, e.g., metabolite and transcript data (Fernie and Stitt, 2012). As the underlying hypothesis about co-regulation for candidate gene finding relies on the fact that transcript levels can serve as a proxy for protein level and that the encoded proteins would interact, we should expect even more powerful approaches once more and more complete proteomic data becomes publicly available.

Unfortunately, these co-regulation approaches rely on close to full genomic coverage, which is currently still difficult to achieve in proteomic sciences despite many promising developments in the last few years (Heazlewood, 2011). As a consequence, it might help to better integrate the proteomic data at hand with data from other “omics” disciplines to facilitate the best use of the data that can be produced now. Indeed some laboratories started generating data-sets comprising more than one “omics” discipline to answer specific biological questions. These integrative approaches have already led to the identification of new target genes and have enabled studies on certain pathways (for an overview, e.g., Tohge et al., 2005; Yonekura-Sakakibara et al., 2008; Mounet et al., 2009; Baginsky et al., 2010; Hannah et al., 2010). However, whilst this is a promising approach, a multitude of resources is necessary for proper data integration, analysis, and interpretation.

Data integration is one of the specialties of the Gabi Primary Database (GabiPD^1^ Riaño-Pachón et al., 2009). As such, GabiPD constitutes a repository and analysis platform for a wide array of heterogeneous data in different plant species. Its strength is the extensive underlying sequence information that helps not only to integrate between the different “omics” disciplines, but also to bridge between different plant species. Therefore, currently one major way to access data is in a gene- or protein-centric way, where data can be accessed based on sequence similarity, keywords, or simply identifiers. It is then possible to link to other data resources.

## ACCESS TO PLANT PROTEOMIC DATA IN GabiPD

The plant proteomic data that is being hosted by GabiPD is available on a specific proteomics microsite^2^. The proteomics pages provide access to annotated 2D-PAGE gel images from *Arabidopsis thaliana* and *Brassica napus* (see below), to a new *Arabidopsis* subcellular protein prediction engine,**and to the Phospho-proteomics page^3^.

Phosphoproteomics is presented as a collection of potential protein kinase substrates of different mitogen-activated protein kinases (MAPKs) and MAPK kinases (MAPKKs) in *Arabidopsis* which were derived from protein microarray experiments (see below).

Furthermore, the GabiPD plant proteomics portal serves as a knowledge repository by providing an overview over several important publications and links to other plant proteomics resources and groups. Thus, it is possible for researchers which are new to proteomics to get a quick overview of this emerging field.

## SUBCELLULAR PREDICTION BASED ON EXPRESSION DATA

The last few years have seen a major improvement of our knowledge about the subcellular localization of proteins based on meticulously conducted proteomics experiments. Despite this wealth of information, there is no experimentally determined subcellular localization for more than half of all *Arabidopsis* proteins and even less information is available for crop plants.

Therefore, prediction of protein subcellular localization remains a necessary stop-gap. Often, this has been done by identifying signal peptides or by analyzing the protein composition in each compartment (see Emanuelsson et al., 2007 for an overview of these methods). That said, we have recently established that large scale transcript expression might help in predicting the subcellular localization of proteins targeted to the chloroplast. Whilst so far there is only direct evidence for the model plant *Arabidopsis*, transcript expression seems to contain information about the targeting of rice proteins to plastids as well (Ryngajllo et al., 2011). Based on the microarray experiments that were most important for the prediction, it seems likely that protein targeting to the chloroplast is, in this case, based upon strong coordination of chloroplastic processes driven by the light regime or diurnal/circadian cycles (Ryngajllo et al., 2011). Expression also seems to contain some information about mitochondrial localization, however it is not yet clear whether this is also driven by certain mitochondrial processes. Based on the above mentioned findings, we developed SLocX to perform subcellular predictions in *Arabidopsis* (Ryngajllo et al., 2011). In the case of AT1G16000, e.g., we could show that, GFP studies confirmed mitochondrial localization, as predicted by SLocX, despite an apparent absence of an N-terminal import signal. This underlines that SLocX might help in identifying proteins targeted by non-classical pathways.

We had initially provided a separate SLocX web resource^4^ to perform these predictions, this resource is now also integrated into the GreenCards view. In addition, the prediction engine now links back to the Gabi Primary Database, so that users can further benefit from the extensive sequence data presented there.

## ANNOTATED 2DE GELS LINKED WITH GENE-CENTRIC INFORMATION

The efficient separation, visualization, and identification of complex protein populations are prerequisites for successful proteome analysis. 2D electrophoresis (2DE) and subsequent mass spectrometry (MS) to identify individual spots are classical approaches fulfilling these requirements.

GabiPD hosts plant 2DE data providing annotated 2DE images of eight different *Arabidopsis thaliana* tissues and of the 80S ribosome (Giavalisco et al., 2005a,b) as well as of *Brassica napus* phloem and xylem (Kehr et al., 2005; Giavalisco et al., 2006). The* Arabidopsis thaliana* proteins were analyzed by matrix assisted laser desorption/ionization time of flight MS peptide mass fingerprinting (Giavalisco et al., 2005a,b) whereas the *Brassica napus* proteins were identified by MS/MS (tandem MS in an electrospray ionization quadrupole time-of-flight tandem mass spectrometer) followed by database searches resulting in peptide fragmentation spectra (Kehr et al., 2005; Giavalisco et al., 2006). In the case of the *Arabidopsis* resource, the tissue-specific 2DE images include more than 650 different proteins represented by a few thousand spots. Whilst there are obviously fewer proteins for the 80S ribosome, the data is also linked to the underlying graphical Mascot reports, allowing the user to verify the obtained results. In the case of *Brassica napus*, proteomics data for the xylem and phloem sap is available, featuring about 70 and 140 proteins, respectively.

**FIGURE 1:**
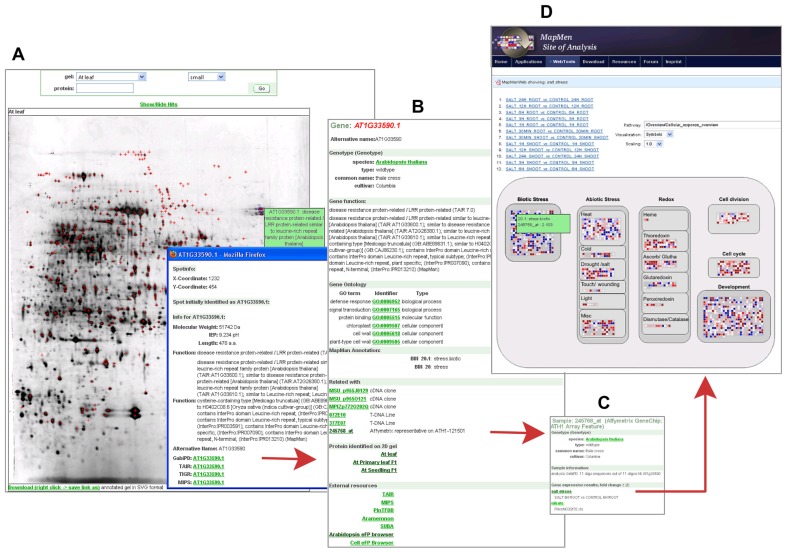
** Proteomic data in GabiPD.** **(A)** 2D-PAGE gel image from *A.thaliana* primary leaf at GabiPDs Proteomics pages. All protein spots identified by MS are highlighted with red crosses. Clicking on one distinct spot (blue cross-hair) directs the user to a more detailed description of the related protein, i.e., a disease resistance protein-related (AT1G33590.1). The related Gene GreenCard is accessible via the integrated GabiPD link. **(B)**Gene GreenCard of AT1G33590.1 with detailed annotation information (GO, MapMan, etc.) and information on all GabiPD data related to this gene, such as links to 2D PAGE-images of other *A. thaliana* tissues. The integrated link to a related Affymetrix probe-set (245768_at) directs the user to the GreenCard of this probe. **(C)** The GreenCard of the Affymetrix probe-set 245768_at includes, beside the probe description, a list of related transcriptomic experiments where the transcript is significantly up- or down-regulated, e.g., a salt stress experiment. The related experimental data are linked. **(D)** MapManWeb user interface at the MapMan Site of Analysis displaying the results of the salt stress transcriptomic experiment in *A. thaliana*. AT1G33590.1 is up-regulated as indicated by a blue filled rectangle representing 245768_at in the “Biotic Stress” field of the presented “Cellular_Response_overview.”

All annotated 2DE images in GabiPD are downloadable in SVG format. This allows the users to obtain a local interactive copy of these images. Thus, the user is able to click on individual spots and to obtain their description as if these images were available online. Moreover, the underlying data is available as an Excel table allowing the direct comparison across the different tissues. In the web resource, the annotated images are searchable by AGI codes or GenBank protein accession codes. As a result, all images including spots of the query protein will be listed and the protein is highlighted by a cross in each image. Protein spots on the gel image are linked with the related Gene GreenCards and *vice versa* to connect proteomic data with gene-centric views. As an example, **Figure 1A** presents a 2DE gel image of *Arabidopsis* leaf. AT1G33590.1, a protein annotated as “disease resistance protein-related” was identified among many other proteins in this gel. From the protein spot, the related Gene GreenCard (**Figure 1B**) is accessible, where gene-centric information is integrated, thus supporting functional annotation. Beside all sequences related to AT1G33590.1, MapMan- (Usadel et al., 2009b) and GO-classification (Ashburner et al., 2000), information on conserved protein domains and orthologs are accessible. The provided MapMan classifications (Usadel et al., 2009b) allow the user to get a quick insight into the potential biological function of the underlying protein, as the MapMan ontology was specifically tailored to plants and has been designed to be as redundancy free as possible.

Within the Gene GreenCard of AT1G33590.1, the user will find links to all other GabiPD data entries related to this gene, including additional 2D gel images of other tissues where the protein is present (**Figure 1B**). In this case, the GreenCard entry indicates that the protein was also identified in primary leaf and seedlings. These tissue-specific protein expression data can be compared to transcript expression data accessible via the gene-specific link to the *Arabidopsis* eFP browser (Winter et al., 2007) in the Gene GreenCard (external links). Transcript expression data are also accessible via the links to Affymetrix representatives on ATH1-121501 that are integrated in the Gene GreenCards. In the case of AT1G33590.1, this is measured by a particular Affymetrix probe-set (245768_at, **Figure 1B**) which directs the user to the sample description (**Figure 1C**) including a list of related transcriptomic experiments where the transcript is up- or down-regulated. AT1G33590.1 is up-regulated, e.g., during salt stress (**Figure 1C**). The whole stress experiment can be visualized in its entirety using the MapManWeb user interface integrated into GabiPD (**Figure 1D**).

## PROTEIN KINASE – SUBSTRATE RELATIONS ON GabiPD’s PHOSPHOPROTEOMICS PAGE

Phosphoproteomics comprises the identification of phosphoproteins, the precise mapping and quantification of phosphorylation sites, and the linkage of phosphorylation sites in substrates to specific protein kinases, which may phosphorylate special amino acid residues under specific physiological conditions (Kersten et al., 2009). Despite recent progress that has been made in the quantitative and dynamic analysis of mapped phosphorylation sites in plants (Schulze, 2010; Novakova et al., 2011), only a handful of plant studies were successful in establishing links between substrates (or even individual phosphorylation sites in a substrate) and a specific protein kinase *in vivo* (in *Arabidopsis*, e.g., Liu and Zhang, 2004; Joo et al., 2008; Lampard et al., 2008; Merkouropoulos et al., 2008; Bethke et al., 2009; Wang et al., 2010; Mao et al., 2011).

**FIGURE 2:**
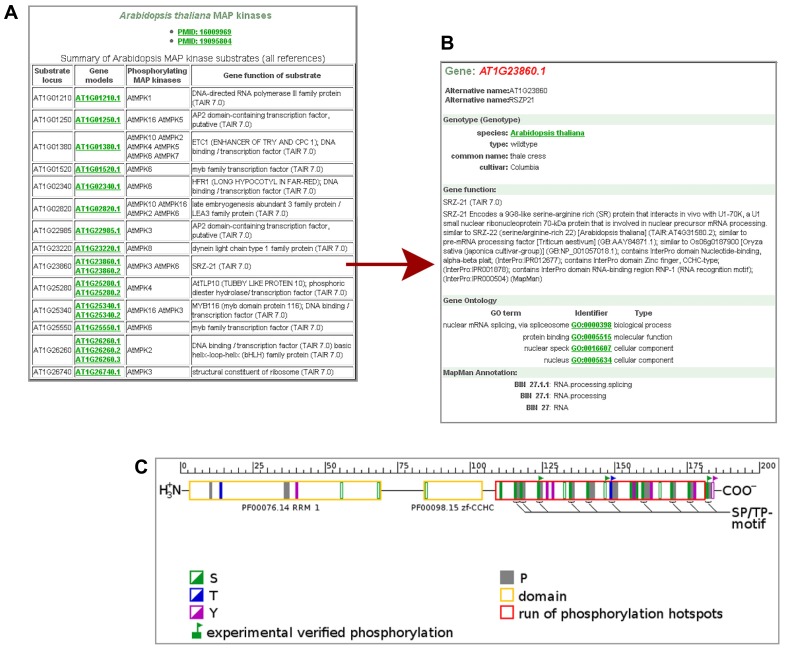
** Phosphoproteomic data in GabiPD.** **(A)** List of potential MAP kinase substrates at GabiPD’s Phosphoproteomics page (www.gabipd.org/projects/Arabidopsis_Proteomics/phosphoproteomics_summary.shtml). Substrates were identified by *in vitro* kinase assays on *Arabidopsis* protein microarrays. AGI codes of the substrates are linked to the related Gene GreenCard in GabiPD. **(B) **Gene GreenCard of RSZP21 with integrated kinase assay result. **(C) **Predicted (filled rectangles in green, blue, and purple) and experimentally verified (flagged rectangles) phosphorylation sites in RSZP21 according to PhosPhAt (Durek et al., 2010; see external links at the Gene GreenCard). The red long box at the C-terminus of the RSZP21 represents a hot spot of phosphorylation predicted recently (Riaño-Pachón et al., 2010). The yellow boxes display conserved protein domains.

Whereas a few plant databases host phosphoproteomic data from medium to large scale studies on protein phosphorylation by MS [e.g., PhosPhAt (Durek et al., 2010), P3DB (Gao et al., 2009), RIPP-DB (Nakagami et al., 2010)], GabiPD provides data on potential protein kinase–substrate relations. These data were taken from different *in vitro* studies based on kinase assays on *Arabidopsis* protein microarrays (Feilner et al., 2005; Popescu et al., 2009). All AGI codes of the potential *Arabidopsis* MAPKK/MAPK substrates identified in these studies, are listed together with their phosphorylating protein kinase(s) and their predicted functions at the Phosphoproteomics pages (**Figure 2A**). The user can switch from the AGI code of a substrate of interest to the related Gene GreenCard, as presented in **Figure 2B** for one of the potential substrates of MPK3 and MPK6, for RSZP21 (AT1G23860.1). The *in vitro*-kinase assay results as well as links to the Gene GreenCard of the phosphorylating kinases are provided here. RSZP21 has been annotated by MapMan to be involved in “RNA.processing/splicing” (**Figure 2B**). This is consistent with results from a large-scale analysis of protein phosphorylation in *Arabidopsis* which has led to the suggestion that the plant mRNA splicing machinery is a major target of phosphorylation (de la Fuente van Bentem et al., 2006).

The user can inspect predicted and experimentally verified phosphorylation sites identified in RSZP21 *in vivo*, when switching to PhosPhAt (Durek et al., 2010) via the external links section at the Gene GreenCard. Although five amino acid residues in RSZP21 have been shown to be phosphorylated in different experiments (**Figure 2C**), so far no link between any one of the phosphorylation sites to a phosphorylating MAPK has been established *in vivo*. Of special interest are SP/TP motifs, because they have been shown to be a consensus motif of MAPK phosphorylation (Bardwell, 2006; Kersten et al., 2009). All of the 10 SP/TP sites of RSZP21 were predicted/experimentally proved to be phosphorylated (**Figure 2C**). Most of the (predicted) phosphorylation sites of RSZP21 are located in a hot spot of phosphorylation that was predicted outside the conserved protein domains at the C-terminus of the protein (red long box in **Figure 2C**; Riaño-Pachón et al., 2010). All these data place RSZP21 on a short-list of top candidate proteins for further *in vivo* verification of their phosphorylation by MAP kinases.

GabiPD’s phosphoproteomics page thus is a valuable source for selecting more substrates for *in vivo *verification. *In vivo* phosphorylation by specific MAPKs of a few potential substrates listed here was already reported, as for ACS-6 (AT4G11280.1; Liu and Zhang, 2004), ERF104 (AT5G61600.1; Bethke et al., 2009), NIA-2 (AT1G37130.1; Wang et al., 2010). Moreover, the substrate list is a great resource for *in silico* approaches for studying cross-talk of different kinases associated in diverse biological processes with their interacting kinases, as recently shown (Taj et al., 2011). Furthermore, this rich resource might represent a good training set for the *in silico* prediction of MAPK-specific phosphorylation site motifs and of MAPK docking sites.

## OUTLOOK

The further development of the proteomics resources in GabiPD will be focused on the extension of the protein kinase–substrate resource to support the discovery of signaling networks in plants. We will annotate plant protein kinases through the MapMan framework. The existing resource on *in vitro* protein kinase–substrate relations will be extended by *in vivo* data. The integration of public data on protein–protein interactions and co-expression will ease the selection of interesting protein kinase–substrate relations from the *in vitro* data for further wet lab investigation.

## Conflict of Interest Statement

The authors declare that the research was conducted in the absence of any commercial or financial relationships that could be construed as a potential conflict of interest.

## Footnotes

1 www.gabipd.org

2 http://www.gabipd.org/projects/Arabidopsis_Proteomics/

3 http://www.gabipd.org/projects/Arabidopsis_Proteomics/phosphoproteomics_summary.shtml

4 http://mapman.mpimp-golm.mpg.de/general/slocx/
